# National survey of blood pressure management practices in peritoneal dialysis

**DOI:** 10.1186/s12882-026-05201-8

**Published:** 2026-07-17

**Authors:** Kashvi Gupta, Chi-yuan Hsu

**Affiliations:** Division of Nephrology, University of California, MUW418 Box 0532, 500 Parnassus Avenue, San Francisco, CA USA

**Keywords:** Blood pressure, Hypertension, Peritoneal dialysis

## Abstract

**Background:**

Contemporary practices for blood pressure (BP) management in peritoneal dialysis (PD) remain poorly characterized despite cardiovascular disease and hypertension being highly prevalent in patients on PD.

**Methods:**

We conducted a national survey of nephrologists in the United States with publicly available email addresses affiliated with the 150 ACGME-accredited adult nephrology training programs. Email invitations were sent to 1786 nephrologists between January and February 2026. 100 nephrologists actively caring for patients on PD consented and completed the survey.

**Results:**

We found wide variation in BP management practices amongst nephrologists. 68% nephrologists use both home and clinic BP measurements to guide treatment decisions, while 32% rely primarily on home BP. Involvement in patient education regarding BP measurement technique was inconsistent. About 25% nephrologists had minimal or no involvement, and 48% had partial involvement with staff-led education. Knowledge about the patient’s home BP device or cuff placement was also limited. The target systolic BP used by nephrologists for patients on PD varied, ranging from < 110 to < 160 mmHg.

**Conclusions:**

Although clinic BP has served as the primary basis for epidemiologic studies linking BP with adverse outcomes in PD, real-world practice appears to depend heavily on home BP measurements. This reliance occurs despite variable oversight of home BP measurement technique among US nephrologists caring for patients on PD. These findings highlight a disconnect between the evidence base and clinical practice. Prospective studies are needed to determine whether home BP–guided management improves outcomes and to define optimal BP targets in PD.

**Supplementary Information:**

The online version contains supplementary material available at https://doi.org/10.1186/s12882-026-05201-8.

## Background

Hypertension is highly prevalent among patients with end-stage kidney disease (ESKD) receiving peritoneal dialysis (PD), and these patients are at elevated risk for cardiovascular disease (CVD) complications and death [1–3]. In the general population, treating hypertension reduces rates of CVD events and mortality. Optimal management of blood pressure (BP) is thus an important component of care in PD, and the approach should account for the unique aspects of care delivery in this patient population. For example, home BP measurements are routinely done by patients on PD [1–5]. 

The International Society of Peritoneal Dialysis Guidelines recommend treating BP to < 140/90 mmHg and that patients check their BP at home at least once a week and at every clinic visit [3]. However, little is known about contemporary practice patterns among nephrologists regarding BP management in patients receiving PD. We conducted a national survey of nephrologists in the United States (U.S.) to fill this knowledge gap.

## Methods

We attempted to reach all nephrologists affiliated with the 150 Accreditation Council for Graduate Medical Education (ACGME) adult nephrology training programs in the U.S. We used this sampling frame because we could potentially systematically obtain their email addresses online (vs. it would be more challenging to obtain such information for private practice nephrologists) and because their practice patterns and opinions are important, as they train the next generation of nephrologists. Through the public-facing websites of these nephrology training programs, email addresses for 1,768 nephrologists were obtained.

The survey instrument was developed after review of relevant literature and guidelines on BP management in PD. Survey items were designed to assess clinically relevant domains, including BP measurement setting, confidence in BP measurements, home BP monitoring practices, physician involvement in BP education, and BP treatment targets. Before national dissemination, we conducted two pilot tests of the survey. In the first pilot test, the survey text was distributed to four UCSF nephrology faculty to assess content validity, face validity, and the clarity of the survey items. In the second pilot test, the survey was formatted in Qualtrics and sent to nine other UCSF nephrology faculty to assess survey flow, skip logic for questions that had linked responses, and gather additional feedback on survey items. All UCSF nephrologists were excluded from the final national sampling frame, and pilot respondents were not included in the analytic sample.”

The final anonymous 5-minute online survey (see Supplement) was emailed via Qualtrics, with three reminder emails sent approximately one week apart between January 22 and February 10, 2026. The survey began by asking for informed consent and whether the respondent cared for ESKD patients on PD. Only those who answered affirmatively were invited to proceed.

Because it was unknown whether nonrespondents were actively caring for patients on PD, the true response rate among eligible nephrologists caring for patients on PD among the 1,768 invited nephrologists could not be directly calculated. We therefore report response metrics using the total number of invited nephrologists as the denominator and the proportion of consenting respondents who actively care for patients receiving PD.

Survey responses were analyzed descriptively, with categorical variables reported as counts and percentages. Optional free-text responses were reviewed narratively, grouped by survey item, and tabulated in the Supplement to provide additional context for the closed-ended responses.

The University of California, San Francisco Institutional Review Board certified this study as exempt research (IRB #25-45625). Online informed consent was obtained from all participants before they started the survey.

## Results

We had 176 respondents to the survey. Of these, 3 did not consent to the survey, 63 who consented but reported not actively caring for patients receiving PD, and 10 who did not complete the survey. Thus, we obtained consent from 173 out of 1,786 (9.68%) nephrologists. Of these, 100 (57.80%) were actively caring for patients receiving PD and constituted the final analytic sample. All respondents in the analytic sample completed the survey in its entirety.

Respondents represented a range of PD clinical experience and practice settings. Most cared for 15 or fewer patients on PD, including 31% who cared for 1–5 patients and 42% who cared for 6–15 patients; 13% cared for 15–30 patients, and 14% cared for more than 30 patients. Respondents also varied in duration of independent nephrology practice caring for patients receiving PD: 14% began practice in 1990 or earlier, 12% between 1991 and 2000, 17% between 2001 and 2010, 41% between 2011 and 2020, and 16% between 2021 and 2025. Most respondents practiced in an academic medical center or university hospital (97%), while some also reported practicing in a dialysis organization (13%), VA or other government hospital (7%), private practice (3%), or healthcare organization/HMO (1%).

Substantial heterogeneity in BP measurement practices and BP target selection was observed among nephrologists (Table 1 and Supplemental Table 1). Most respondents use both home and clinic BP measurements to guide treatment (68%). Diastolic Blood Pressure (DBP) was not a treatment target for half the respondents. The most common Systolic Blood Pressure (SBP) target was < 130 mmHg, but respondents chose targets ranging from < 110 mmHg to < 160 mmHg. Some commented that the “target BP is unknown” in PD.

**Table 1:** Summary of survey responses from nephrologists actively treating peritoneal dialysis (PD) patients (n = 100)

Q1	Approximately how many patients receiving PD do you care for? (n = 100)	
	1–5 patients	31 (31%)
	6–15 patients	42 (42%)
	15–30 patients	13 (13%)
	> 30 patients	14 (14%)
**Q2**	**When managing BP in patients receiving PD, which BP measurement setting do you primarily use to guide treatment decisions? (n = 100)**	
	Home BP measurements	32 (32%)
	PD clinic BP measurements	0 (0%)
	Both Home and PD clinic BP measurements	68 (68%)
**Q3A**	**How confident are you in the accuracy of BP measurements obtained at home (n = 100)**:	
	< 25%	0 (0%)
	25–49%	4 (4%)
	50–74%	24 (24%)
	75–100%	72 (72%)
**Q3B**	**How confident are you in the accuracy of BP measurements obtained at clinic (n = 100)**:	
	< 25%	1 (1%)
	25–49%	10 (10%)
	50–74%	36 (36%)
	75–100%	53 (53%)
**Q4**	**How often do you recommend that your patients receiving PD measure their BP at home? (n = 100)**	
	Daily	61 (61%)
	Multiple times per day	13 (13%)
	Several times per week	14 (14%)
	Weekly	4 (4%)
	Other	8 (8%)
**Q5**	**How involved are you in your patients’ BP education during PD training? (n = 100)**	
	Minimally or not involved – I have little or no direct involvement in education	25 (25%)
	Somewhat involved – I provide some education, majority staff-led	48 (48%)
	Very involved – I personally provide all or most of the education	26 (26%)
	Other	1 (1%)
**Q6**	**For your patients receiving PD, how often do you know which BP device (brand or cuff placement) they use at home? (n = 100)**	
	Always	12 (12%)
	Often	22 (22%)
	Sometimes	31 (31%)
	Rarely/Never	35 (35%)
**Q7**	**What is your home SBP goal in patients receiving PD? (n = 100)**	
	Less than 110 mmHg	1 (1%)
	Less than 120 mmHg	16 (16%)
	Less than 130 mmHg	59 (59%)
	Less than 140 mmHg	23 (23%)
	Less than 150 mmHg	0 (0%)
	Less than 160 mmHg	1 (1%)
**Q8**	**What is your clinic SBP goal in patients receiving PD? (n = 68)**	
	Less than 110 mmHg	0 (0%)
	Less than 120 mmHg	5 (7%)
	Less than 130 mmHg	33 (49%)
	Less than 140 mmHg	27 (40%)
	Less than 150 mmHg	2 (3%)
	Less than 160 mmHg	1 (1%)
**Q9**	**Do you target a diastolic blood pressure (DBP) in patients receiving PD? (n = 100)**	
	Yes	50 (50%)
	No	50 (50%)
**Q10**	**What is your DBP goal in patients receiving PD? (n = 50)**	
	50–60 mmHg	1 (2%)
	61–70 mmHg	3 (6%)
	70–80 mmHg	36 (72%)
	80–90 mmHg	9 (18%)
	90–100 mmHg	1 (2%)

Involvement in educating patients on BP measurement technique varied: 25% reported minimal or no involvement, and 48% reported some involvement (mostly staff-led). Only 26% reported personally providing all or most of the BP education. Awareness about the patient’s home BP devices, including cuff placement, was limited: 12% reported always knowing the BP device brand or cuff placement used at home, whereas 35% reported rarely or never knowing this information.

## Discussion

To our knowledge, this is the first study to systematically assess contemporary practice patterns among U.S. nephrologists regarding the management of hypertension in ESKD patients on maintenance PD. Our results reveal that nephrologists rely heavily on home BP readings but raise concerns about the adequacy of physician oversight in training patients to obtain reliable home BP readings. In addition, there was heterogeneity in the BP target.

Greater reliance on home BP measurements is consistent with recent guidelines [2, 4]. However, guidelines also emphasize that home BP should complement, not replace, standardized clinic BP [2]. Physicians relying solely on home BP are therefore not aligned with guideline-based practice, but this was the self-reported practice in 32% of survey respondents. While our study did not directly evaluate adherence to BP measurement protocols or the validity of home BP values in patients receiving PD, our survey findings revealed that nephrologists have variable involvement in BP education and limited routine awareness of patients’ home BP device type or cuff placement. Prior studies have shown that home BP monitoring can be inaccurate in real-world settings because of device-related and patient technique errors. For example, a study of patients seen in a Canadian nephrology clinic found that 30% of patient-owned monitors reported systolic BP values differing from mercury sphygmomanometer readings by ≥ 5 mmHg [6]. A Polish study of 100 hypertensive patients filmed while measuring their BP on their own devices, reported that only 3% patients performed the measurement without error, 60% made three or more errors, and the most frequent error—incorrect cuff placement—occurred in 76% of subjects [7]. Notably, only 29% had received adequate physician instruction on proper technique [7]. A recent scoping review summarizing 17 studies from around the world identified 35 distinct errors committed during home BP monitoring, including inadequate back support (36%) and talking during measurement (36%) [8]. Taken together, these findings suggest that without routine education regarding BP measurement technique and assessment of BP devices and cuff placement, practices that our survey indicates are not consistently performed by nephrologists in PD clinics, the accuracy of home BP readings used to guide treatment decisions cannot be assumed. Inaccurate or non-standardized home BP data may affect decisions regarding volume status, titration of antihypertensive medication, and lost opportunities to reduce rates of cardiovascular disease in this high-risk population.

Given the home-based nature of PD, telemedicine, mobile health applications, and remote patient monitoring are practical strategies to optimize hypertension management and reduce downstream cardiovascular morbidity. Few remote patient monitoring programs in PD have been associated with improved BP control and technique survival, and decreased hospitalization rates [9, 10]. These tools may be particularly useful when paired with multidisciplinary care models involving nurses, pharmacists, and dialysis staff [4, 11]. Although evidence specific to BP management in PD remains limited, studies in cardiovascular populations suggest that digital health interventions and smart-device–based monitoring can improve patient engagement and hypertension-related risk-factor management [12–15]. 

It is notable that no respondent reported relying exclusively on clinic BP to manage hypertension. Yet the epidemiology literature on the association between BP levels and adverse outcomes among PD patients is almost exclusively based on clinic BP readings. For example, of the 30 studies included in a recent meta-analysis by Lee et al., none reported using home BP as the exposure of interest (one used ambulatory blood pressure monitoring). Some studies did not explicitly state where BP was measured, but the vast majority relied on office BP measurements [16]. 

The reasons behind the high level of physician confidence in home BP readings observed in our survey cannot be determined (we did not include this as part of the survey). We speculate this may reflect perceived advantages of home monitoring, including the availability of repeated longitudinal measurements over days to weeks (vs. less frequent clinic measurements), avoidance of the white-coat effect, concerns about suboptimal BP measurement techniques at the PD clinic, and the increasing use of data transmission platforms embedded in PD cyclers that facilitate review of home BP trends. However, physician confidence in home BP readings should not be equated with confirmed measurement accuracy. Because the accuracy of home readings depends on adherence to proper measurement technique [17, 18]. Accordingly, while the growing infrastructure supporting home BP monitoring may reasonably increase physician confidence in these readings, this confidence must be tempered by systematic efforts to ensure measurement quality.

We also observed heterogeneity in SBP targets, with most nephrologists choosing targets ranging from SBP 140 mmHg to 120 mmHg. We suspect that this variation in practice patterns reflects the limited evidence base for optimal BP targets in the PD population and the need to extrapolate from guidelines developed largely for non-dialysis populations. The KDOQI US Commentary on the 2020 ISPD Practice Recommendations noted the paucity of robust evidence underpinning BP target recommendations in PD, yet recommended that this evidence gap should not preclude clinicians from tailoring volume management strategies and BP goals to the individual patient [19]. In our survey, free-text responses indicated that clinicians individualize BP targets based on the patient’s age, frailty, cardiovascular comorbidities, orthostatic symptoms, and concern about hypotension. Thus, heterogeneity in BP targets may also reflect deliberate, patient-centered adjustment of BP goals in a population vulnerable to both hypertension-related cardiovascular complications and hypotension from overtreatment.

The 2021 KDIGO Blood Pressure Guideline recommends a target SBP < 120 mmHg for adults with CKD, largely based on SPRINT data demonstrating cardiovascular benefit from intensive BP lowering [20, 21]. However, this recommendation applies to non-dialysis CKD, and the guideline acknowledges limited evidence for patients receiving dialysis. The 2025 AHA/ACC Hypertension Guideline similarly recommends SBP < 130 mmHg for adults with CKD but explicitly states that these recommendations may not apply to those on dialysis, given limited data in patients receiving chronic hemodialysis or peritoneal dialysis [4]. In contrast, the 2015 ISPD Guidelines recommend a BP target of < 140/90 mmHg in the PD population, a threshold that was extrapolated from older studies in general and nondialysis CKD populations rather than derived from PD-specific trials [3]. The 2020 ISPD Practice Recommendations stated that “there is currently no evidence for a specific blood pressure target in PD (practice point),” [22] and the 2020 KDOQI US Commentary on this noted that some investigators have proposed relaxing BP targets in PD to accept SBP up to 159 mmHg while avoiding hypotension, given observational data showing higher mortality at very low SBP but no clear harm at moderately elevated levels [16, 19, 23]. Thus, findings in our survey reflect the range spanned by these guidelines, and the variation among respondents mirrors the lack of convergence among guideline bodies themselves.

This study has limitations. We distributed the survey only to nephrologists affiliated with U.S. ACGME programs. Therefore, respondents may not reflect nephrologists practicing in non-academic settings, smaller PD programs, or settings with fewer standardized PD resources. In addition, it is possible that respondents may differ from non-respondents in PD practice patterns. Clinicians with a greater interest in PD, hypertension, or home BP monitoring are more likely to participate. The survey response rate was modest, but comparable to other email-based physician survey studies, which reported response rates ranging 5–19% [24, 25]. Because we could not determine how many of the invited nephrologists actively cared for patients receiving PD, we used the response rate among all invited nephrologists instead, which likely underestimates the true response rate amongst eligible nephrologists. Finally, self-reported practices of nephrologists may differ from actual clinical behavior.

## Conclusion

In conclusion, BP management practices among nephrologists caring for patients on PD vary considerably. Nephrologists rely heavily on home BP measurements but report inconsistent attention to patient education for obtaining accurate BP data. These results underscore the immediate need to improve standards of BP measurement in current clinical practice.

## Supplementary Information

Below is the link to the electronic supplementary material.

Supplementary Material 1

Supplementary Material 2

## Data Availability

The datasets used and analyzed during the current study are available from the corresponding author on reasonable request.

## Abbreviations

ACGME: Accreditation Council for Graduate Medical Education
BP: Blood Pressure
CVD: Cardiovascular Disease
DBP: Diastolic Blood Pressure
ESKD: End Stage Kidney Disease
PD: Peritoneal Dialysis
SBP: Systolic Blood Pressure
U.S.: United States

## References

[1] Mayeda L, Bansal N. Hypertension in patients with end-stage kidney disease requiring dialysis: bridging the divide between evidence and practice. Hypertension. 2026-03;83(3). 10.1161/HYPERTENSIONAHA.125.24344 PMC12854527 41603034

[2] Group KDIGOKCW. KDIGO 2024 Clinical Practice Guideline for the Evaluation and Management of Chronic Kidney Disease. Kidney Int. 2024;105(4S):S117–314. 10.1016/j.kint.2023.10.018 38490803

[3] Wang AYM, Brimble KS, Brunier G, Holt SG, Jha V, Johnson DW et al. ISPD Cardiovascular and metabolic guidelines in adult peritoneal dialysis patients part i – assessment and management of various cardiovascular risk factors. Perit Dialysis International: J Int Soc Perit Dialysis. 2015 Jul-Aug;35(4). 10.3747/pdi.2014.00279 PMC4520720 26228782

[4] Jones DW, Ferdinand KC, Taler SJ, Johnson HM, Shimbo D, Abdalla M, et al. 2025 AHA/ACC/AANP/AAPA/ABC/ACCP/ACPM/AGS/AMA/ASPC/NMA/PCNA/SGIM Guideline for the Prevention, Detection, Evaluation, and Management of High Blood Pressure in Adults: A Report of the American College of Cardiology/American Heart Association Joint Committee on Clinical Practice Guidelines. J Am Coll Cardiol. 2025;86(18):1567–678. 10.1016/j.jacc.2025.05.007 40815242

[5] Kramer HJ, Townsend RR, Griffin K, Flynn JT, Weiner DE, Rocco MV, et al. KDOQI US Commentary on the 2017 ACC/AHA Hypertension Guideline. Am J Kidney Dis. 2019;73(4):437–58. 10.1053/j.ajkd.2019.01.007 PMC6740329 30905361

[6] Ruzicka M, Akbari A, Bruketa E, Kayibanda JF, Baril C, Hiremath S. How Accurate Are Home Blood Pressure Devices in Use? A Cross-Sectional Study. PLoS ONE. 2016;11(6):e0155677. 10.1371/journal.pone.0155677 PMC4889144 27249056

[7] Nessler K, Krztoń-Królewiecka A, Suska A, Mann MR, Nessler MB, Windak A. The quality of patients’ self-blood pressure measurements: a cross-sectional study. BMC Cardiovasc Disord. 2021;21(1):539. 10.1186/s12872-021-02351-5 PMC8588592 34772348

[8] Cavalcante FML, Brito OD, Estácio ACS, Kumakura ARSO, Chaves AFL, Galindo Neto NM, et al. Errors and negative outcomes in home blood pressure monitoring by hypertensive individuals: a scoping review. Blood Press Monit. 2026;31(1):6–13. 10.1097/MBP.0000000000000778 41186210

[9] Biebuyck GKM, Neradova A, de Fijter CWH, Jakulj L. Impact of telehealth interventions added to peritoneal dialysis-care: a systematic review. BMC Nephrol. 2022;23(1):292. 10.1186/s12882-022-02869-6 PMC9396599 35999512

[10] Yeter HH, Manani SM, Ronco C. The utility of remote patient management in peritoneal dialysis. Clin Kidney J. 2021;14(12):2483–9. 10.1093/ckj/sfab111 PMC8344514 34938532

[11] Tucker KL, Sheppard JP, Stevens R, Bosworth HB, Bove A, Bray EP, et al. Self-monitoring of blood pressure in hypertension: A systematic review and individual patient data meta-analysis. PLoS Med. 2017;14(9):e1002389. 10.1371/journal.pmed.1002389 PMC5604965 28926573

[12] Hayıroğlu M, Çınar T, Cilli Hayıroğlu S, Şaylık F, Uzun M, Tekkeşin A. The role of smart devices and mobile application on the change in peak VO. Acta Cardiol. 2023;78(9):1000–5. 10.1080/00015385.2023.2223005 37318090

[13] Şaylık F, Çınar T, Hayıroğlu M, Tekkeşin A. Digital Health Interventions in Patient Management Following Acute Coronary Syndrome: A Meta-Analysis of the Literature. Anatol J Cardiol. 2023;27(1):2–9. 10.14744/AnatolJCardiol.2022.2254 PMC9893709 36680440

[14] Hayıroğlu M. Telemedicine: Current Concepts and Future Perceptions. Anatol J Cardiol. 2019;22(Suppl 2):21–2. 10.14744/AnatolJCardiol.2019.12525 31670712

[15] Abdalla M, Bolen SD, Brettler J, Egan BM, Ferdinand KC, Ford CD, et al. Implementation Strategies to Improve Blood Pressure Control in the United States: A Scientific Statement From the American Heart Association and American Medical Association. Hypertension. 2023;80(10):e143–57. 10.1161/HYP.0000000000000232 PMC10578150 37650292

[16] Lee V, Siami H, Peschard VG, Malekinejad M, Huang E, Tahir P, et al. Blood Pressure Level and Risk of All-Cause Mortality in Patients With Kidney Failure on Maintenance Peritoneal Dialysis: A Systematic Review and Meta-Analysis of Observational Studies. Kidney Med. 2026;8(2):101193. 10.1016/j.xkme.2025.101193 PMC12864663 41640791

[17] Viera AJ, Yano Y, Lin FC, Simel DL, Yun J, Dave G, et al. Does This Adult Patient Have Hypertension? The Rational Clinical Examination Systematic Review. JAMA. 2021;326(4):339–47. 10.1001/jama.2021.4533 34313682

[18] Shimbo D, Artinian NT, Basile JN, Krakoff LR, Margolis KL, Rakotz MK, et al. Self-Measured Blood Pressure Monitoring at Home: A Joint Policy Statement From the American Heart Association and American Medical Association. Circulation. 2020;142(4):e42–63. 10.1161/CIR.0000000000000803 32567342

[19] Teitelbaum I, Glickman J, Neu A, Neumann J, Rivara MB, Shen J, et al. KDOQI US Commentary on the 2020 ISPD Practice Recommendations for Prescribing High-Quality Goal-Directed Peritoneal Dialysis. Am J Kidney Dis. 2021;77(2):157–71. 10.1053/j.ajkd.2020.09.010 33341315

[20] Wright JT, Williamson JD, Whelton PK, Snyder JK, Sink KM, Rocco MV, et al. A Randomized Trial of Intensive versus Standard Blood-Pressure Control. N Engl J Med. 2015;373(22):2103–16. 10.1056/NEJMoa1511939 PMC4689591 26551272

[21] Cheung AK, Chang TI, Cushman WC, Furth SL, Hou FF, Ix JH, et al. Executive summary of the KDIGO 2021 Clinical Practice Guideline for the Management of Blood Pressure in Chronic Kidney Disease. Kidney Int. 2021;99(3):559–69. 10.1016/j.kint.2020.10.026 33637203

[22] Brown EA, Blake PG, Boudville N, Davies S, de Arteaga J, Dong J, et al. International Society for Peritoneal Dialysis practice recommendations: Prescribing high-quality goal-directed peritoneal dialysis. Perit Dial Int. 2020;40(3):244–53. 10.1177/0896860819895364 32063219

[23] Bezerra R, Teles F, Nadruz W, Feitosa ADM, Coelho JAPM, Ponce D, et al. Association between blood pressure and cardiovascular events and mortality in patients on peritoneal dialysis: a systematic review and meta-analysis of observational studies. Clin Exp Nephrol. 2025;29(12):1840–9. 10.1007/s10157-025-02719-3 40545494

[24] Kim B, Ryan K, Kasun M, Roberts LW, Kim J. Online survey assessing US primary care physicians’ attitudes toward AI use in clinical administrative tasks. BMJ Health Care Inf. 2026;33(1). 10.1136/bmjhci-2024-101400 PMC12815081 41513291

[25] Sinsky CA, Dyrbye LN, West CP, Satele D, Tutty M, Shanafelt TD. Professional Satisfaction and the Career Plans of US Physicians. Mayo Clin Proc. 2017;92(11):1625-35. 10.1016/j.mayocp.2017.08.017 29101932

