# Supplementary material for: National survey of blood pressure management practices in peritoneal dialysis
Source: BMC Nephrol. 2026 Jul 17;27:613. doi: 10.1186/s12882-026-05201-8 (PMC13670639; doi:10.1186/s12882-026-05201-8)
Supplement: Supplementary file 1 [file 12882_2026_5201_MOESM1_ESM.docx]

Supplement: Blood Pressure Management in Peritoneal Dialysis Survey used in the study.

**University of California, San Francisco**

**Blood Pressure Management in Peritoneal Dialysis**

**Information Sheet and Online Consent:**

You are invited to participate in a research study conducted by Dr. Kashvi Gupta (Nephrology Fellow) and Dr. Chi-yuan Hsu, MD, MSc (Division Chief of Nephrology), at the University of California, San Francisco (UCSF).

If you choose to participate, you will be asked to complete a brief survey. The purpose of this study is to gain clinical insight into current practice patterns for blood pressure management in patients receiving peritoneal dialysis. You were identified as a potential participant because you are a practicing nephrologist at an ACGME-accredited nephrology program in the United States.

The survey will take **less than 5 minutes** to complete.

Participation is entirely voluntary. No personal identifying information will be collected, and all responses will remain anonymous. You may stop participation at any time.

If you have questions about the study, please contact Kashvi Gupta at kashvi.gupta@ucsf.edu. If you have questions or concerns about your rights as a research participant, you may contact the UCSF Institutional Review Board at 415-476-1814.

If you agree to participate in this study, please click the **Agree** button below to begin the survey.

- **Agree**
- **Disagree**

**Section 1: Eligibility**

**1. Do you currently care for adult patients receiving PD?***

- Yes
- No

(If “No,” end of survey.)

**2. Approximately how many patients receiving PD do you care for?*** (Select one)

- 1–5
- 6–15
- 16–30
- >30

**Section 2: Blood Pressure Monitoring Practices**

**3A. When managing BP in patients receiving PD, which BP measurement setting do you primarily use to guide treatment decisions?*** (Select one)

- Home BP measurements
- PD clinic BP measurements
- Both home and PD clinic BP measurements

(Display Q8A–B if “Home BP measurements” is selected; display Q8C–D if “PD clinic BP measurements” is selected; display Q8A–D if “Both” is selected.)

**3B. Please share any comments about the BP measurement setting you use to guide treatment decisions (optional):**

**4. How confident are you in the accuracy of BP measurements obtained at:**

*0 = not at all confident and 100 = very confident*

**Home** (Scale from “Not at all confident” to “Very confident”, responses obtained as 0 to 100 in increments of 25.)

**PD Clinic** (Scale from “Not at all confident” to “Very confident”, responses obtained as 0 to 100 in increments of 25.)

**5. How often do you recommend that your patients receiving PD measure their BP at home?*** (Select one)

- Weekly
- Several times per week
- Daily
- Multiple times per day
- No routine recommendation
- Other (please specify): __________

**6. How involved are you in your patients’ BP education during PD training?*** (Select one)

- Very involved – I personally provide all or most of the education
- Somewhat involved – I provide some education, majority staff-led
- Minimally or not involved – I have little or no direct involvement in education
- Other (please specify): __________

**7. For your patients receiving PD, how often do you know which BP device (brand or cuff placement) they use at home?*** (Select one)

- Always
- Often
- Sometimes
- Rarely or never

**Section 3: Target Blood Pressure**

**8A. What is your target home systolic BP (SBP) in PD patients?***
SBP mmHg (Scale from 90 to 180 mmHg, in increments of 10)

**8B. Please share any comments regarding your home SBP target (optional):**

**8C. What is your target PD clinic systolic BP (SBP) in PD patients?***
SBP mmHg (Scale from 90 to 180 mmHg, in increments of 10)

**8D. Please share any comments regarding your PD clinic SBP target (optional):**

**9A. Do you target a diastolic BP (DBP) in PD patients?*** (Select one)

- Yes
- No

(Display 9B and 9C only if “Yes” selected in 9A, else skip to 10.)

**9B. What is your target diastolic BP (DBP) in PD patients?***
DBP mmHg (Scale from 60 to 100 mmHg, in increments of 10)

**9C. Please share any comments regarding your DBP target (optional):**
*If applicable, indicate if your DBP targets differ for home and PD clinic BP measurements*

**Section 4: Demographics**

**11. In what year did you begin your independent nephrology practice and start caring for patients receiving PD?***
Dropdown: Starting from 1990 or earlier to 2025

**12. What type(s) of medical practice do you work in?***
(Select all that apply)

- Academic medical center/university hospital
- Private practice
- Hospital-employed (non-university)
- Healthcare organization / HMO
- VA / other government hospitals
- Dialysis organization (e.g., DaVita, Fresenius, US Renal, Satellite)
- Industry / Other (please specify): __________

**We thank you for your time spent taking this survey. Your response has been recorded.

If you have any feedback, please email** [**kashvi.gupta@ucsf.edu**](mailto:kashvi.gupta@ucsf.edu)

* Signifies forced response
