# Supplementary material for: National survey of blood pressure management practices in peritoneal dialysis
Source: BMC Nephrol. 2026 Jul 17;27:613. doi: 10.1186/s12882-026-05201-8 (PMC13670639; doi:10.1186/s12882-026-05201-8)
Supplement: Supplementary file 2 [file 12882_2026_5201_MOESM2_ESM.docx]

Supplement Table: Qualitative responses obtained from nephrologists in the survey.

| **Q2** | **When managing BP in patients receiving PD, which BP measurement setting do you primarily use to guide treatment decisions?** | |
| --- | --- | --- |
|  | A | “Daily BP readings built into the Sharesource Platform give us a good idea of the average BP control.” |
|  | B | “More accurate home BP readings could be achieved if the readings were transmitted by Bluetooth technology to a modem bypassing patient entering a real or false reading manually.” |
|  | C | “I look at the monthly average generated by software. I see the maximum and minimum BP.” |
|  | D | “Typically, I am predisposed to favor home BP readings.” |
|  | E | “By review of the daily entries of BP data from home and monthly BP in clinic.” |
|  | F | “Mostly review home BPs on their log.” |
|  | G | “Looking at both environments seems to be instructive of a good average BP reading.” |
|  | H | “Home supersedes clinic measures when they are discrepant.” |
|  | I | “I use home more than clinic BP measurements.” |
|  | J | “I try to maintain systematic measurements from home measured in AM and PM.” |
|  | K | “Symptoms and fluctuations in BP.” |
|  | L | “I compare both. I make sure home equipment is calibrated. I assess carefully for low BP symptoms.” |
|  | M | “Overall, I use home measurements but if they are much different than clinic visits, I investigate as to why.” |
|  | N | “Home BPs are more reliable.” |
|  | O | “< 130 Systolic BP.” |
|  | P | “Home BP measurements are better because there are more, although I do look for big discrepancies.” |
| **Q4** | **How often do you recommend that your patients receiving PD measure their BP at home?** | |
|  | A | “With every PD treatment.” |
|  | B | “5 days before visit, twice a day.” |
|  | C | “Each time they do dialysis.” |
|  | D | “It varies with patient.” |
|  | E | “Depends on stability and whether they have hypertension.” |
|  | F | “Depends on the BP trends.” |
|  | G | “Daily, pre- and post-PD.” |
|  | H | “While on PD.” |
| **Q5** | **How involved are you in your patients' BP education during PD training?** | |
|  | A | “I am not the nephrologist in charge of training. i take care of PD patients after training complete.” |
| **Q7** | **What is your home SBP goal in patients receiving PD? (n=100)** | |
|  | A | “Depends on the patients age, for majority 130/80 mmHg but < 120 mmHg if young (< 50 years). |
|  | B | “< 135 mmHg.” |
|  | C | “Less than or between 130 to 140 mmHg.” |
|  | D | “The ideal BP in end stage kidney disease is unknown.” |
|  | E | “Meaning less than 130 mmHg.” |
|  | F | “Goal is dependent on other factors including age, co-morbidities and diastolic BP.” |
|  | G | “My goal is less than 140 mmHg.” |
|  | H | “< 140 mmHg.” |
|  | I | “This is the goal- there are many aspects that go into the achieved BP.” |
|  | J | “Most patients I have on PD are elderly/frail, with intermittent symptoms with low BPs. Target average BP 120s mmHg.” |
|  | K | “Again, I monitor symptoms and pay attention to the highest and lowest BP. I make adjustment accordingly.” |
|  | L | “The goal is 120-130 mmHg, however, if any concern of autonomic insufficiency causing orthostatic hypotension, then 140 mmHg sitting and 110 mmHg standing.” |
|  | M | “< 130 mmHg.” |
|  | N | “It may vary, sometimes 130-140 mmHg depending on the patient.” |
|  | O | “I aim for SBP < 140 mmHg.” |
|  | P | “As far as I know, there is no guideline specific to PD, so I use the 130 mmHg as my threshold.” |
|  | Q | “Individualized.” |
|  | R | “Typically, < 130/80 mmHg pre-PD but avoiding very low BP.” |
|  | S | “BPs are age determinate and whether patients have coronary artery disease, peripheral artery disease, or claudication.” |
|  | T | “130/80 mmHg” |
|  | U | “Generally, target a range of 110 to 130 mmHg with percentage of values in the target range as a good assessment overall.” |
|  | V | “I aim for 130 mmHg unless it affects their ability to achieve euvolemia.” |
|  | W | “The optimal BP for dialysis patients is not known. I am more aggressive in younger, transplant eligible patients or in those whom I think can tolerate a lower blood pressure.” |
| **Q8** | **What is your clinic SBP goal in patients receiving PD? (n=68)** | |
|  | A | “Frequently higher than target home BP.” |
|  | B | “The target BP is unknown. The BP is only a marker for fluid status.” |
|  | C | “If patients bring in their home cuff as instructed, to verify calibration, and they have proven white coat syndrome, then I believe the home values, not the clinic values.” |
|  | D | “<140 mmHg” |
|  | E | “I allow for some degree of white coat hypertension, but if home BP is high as well then I will be more aggressive.” |
|  | F | “BP in clinic tend to be higher on average.” |
|  | G | “I rely on multiple home BP readings. I almost ignore clinic BP, unless it is extreme. I have no “goal”, I just compared to home BP.” |
|  | H | “< 130 mmHg” |
|  | I | “Same parameters as home BP 130-140 mmHg, depending on the patient.” |
|  | J | “Depending on how close home and clinic readings are…” |
|  | K | “Age dependent and whether other co-morbidities are present.” |
|  | L | “Taking into account concordance with home values and also assuming no orthostasis, etc.” |
| **Q9** | **What is your DBP goal in patients receiving PD? (n=50)** | |
|  | A | “Goal less than 100 mmHg.” |
|  | B | “Less than.” |
|  | C | “Low DBP can limit achieved SBP particularly in older patients.” |
|  | D | “As per SBP, but DBP is rarely an issue, except in younger patients. |
|  | E | “< 90 mmHg.” |
|  | F | “I worry a lot about hypotension.” |
|  | G | “DBP is more of a safety signal for older patients to avoid overtreatment.” |
| Abbreviations: PD, peritoneal dialysis; BP, blood pressure; SBP, systolic blood pressure; DBP, diastolic blood pressure. | | |
